# Facilitators and barriers to participation in prehabilitation prior to orthopaedic elective surgery – a qualitative study with elderly (pre-)frail patients

**DOI:** 10.1186/s12877-025-06592-3

**Published:** 2025-11-04

**Authors:** Carina Schöne, Tamina I. Fuchs, Jörn Kiselev, Katrin Schmidt, Stefan J. Schaller, Claudia Spies, Tanja Rombey

**Affiliations:** 1 Berlin School of Public Health, Charité – Universitätsmedizin Berlin, Berlin, Germany; 2 Federal Institute for Occupational Safety and Health, BAuA, Berlin, Germany; 3 Department for Anesthesiology and Intensive Care Medicine (CCM/CVK), Charité – Universitätsmedizin Berlin, Corporate Member of Freie Universität Berlin, Humboldt-Universität zu Berlin, Berlin Institute of Health, Berlin, Germany; 4 Department for Health Sciences, Hochschule Fulda University of Applied Sciences, Fulda, Germany; 5 Department of Anaesthesia, Intensive Care Medicine and Pain Medicine, Clinical Division of General Anaesthesia and Intensive Care Medicine, Medical University of Vienna, Vienna, Austria; 6 Department of Health Care Management, Technische Universität Berlin, Straße des 17. Juni 135, 10623 Berlin, Germany

**Keywords:** Frailty, Prehabilitation, Facilitators, Barriers, Implementation, Qualitative research

## Abstract

**Background:**

Patients with frailty syndrome have limited functional resources and an increased risk of postoperative complications. Prehabilitation may reduce adverse events and improve postoperative recovery. This study aimed to investigate the facilitators and barriers to participation in prehabilitation prior to elective surgery from the perspective of elderly (pre-)frail patients.

**Methods:**

This qualitative study was nested within the PRAEP-GO trial, a multicentre randomised controlled trial currently investigating the (cost-)effectiveness of multimodal prehabilitation for elderly (pre-)frail patients in Germany. From February to April 2023, semi-structured face-to-face interviews were conducted with patients allocated to the intervention group of the PRAEP-GO trial. After verbatim transcription of the interviews, data were analysed using content-structuring qualitative content analysis. Results were synthesised using the capability, opportunity, motivation and behaviour (COM-B) model.

**Results:**

Eight patients with a mean age of 78.5 ± 3 years were interviewed in their homes. All interviewees had an indication for an orthopaedic surgery and had participated in the prehabilitation programme offered within the PRAEP-GO trial. In total, 32 facilitators and 25 barriers to participation were identified. These could be assigned to the entire spectrum of the COM-B model. The focus on individual needs, personalised advice from healthcare professionals and a well-organised prehabilitation programme facilitated participation in prehabilitation. Gaps in knowledge, limited physical functioning, (co-)morbidities and an inflexible attitude were identified as barriers.

**Conclusions:**

These insights from the elderly (pre-)frail patient’s perspective are an essential addition to the quantitative data generated in prehabilitation trials. The identified facilitators and barriers should be considered in the future implementation of prehabilitation programmes for this population.

**Trial registration:**

PRAEP-GO trial: ClinicalTrials.gov (NCT04418271; June 1, 2020), Qualitative study: Open Science Framework Registries (osf.io/xnbqc; February 18, 2023).

**Supplementary Information:**

The online version contains supplementary material available at 10.1186/s12877-025-06592-3.

## Introduction

In an era of increasing life expectancy and medical advances, a growing number of older people are undergoing major and complicated surgical procedures each year. In Germany, approximately one-third of all surgeries are performed on people aged 70 and over [1]. With increasing age, the likelihood of postoperative complications and death following surgery also increases [2–4]. People with frailty syndrome are particularly affected by postoperative complications, medium- and long-term limitations, increased mortality and prolonged hospitalisation [4–6]. Thus, pre-operative frailty screening to consider specific risk factors in this population is highly recommended [7].

Fried et al. 2001 [8] defined frailty as a reversible clinical syndrome characterised by the following features: unintentional weight loss, self-reported fatigue, low physical activity, slow walking speed and reduced grip strength. The presence of one or two of these characteristics is labelled prefrailty, while frailty is defined as the presence of three or more symptoms [8]. In Germany, the prevalence of frailty in people aged 65 years and older is estimated at 14% and the prevalence of prefrailty at 40% [9]. It is, therefore, essential to offer patients with (pre-)frailty the opportunity to regain the highest possible level of function after surgery [10].

Multimodal prehabilitation before elective surgery aims to optimise patients’ preoperative functioning [3, 11, 12] primarily to reduce the risk of adverse postoperative outcomes or negative progression and to support rehabilitation [11, 13]. Though following a different approach, multimodal prehabilitation includes similar modalities as rehabilitation [7, 14], e.g., physiotherapy or exercise therapy, psychological counselling, occupational therapy, speech therapy, as well as nutritional and social counselling. However, the components, duration, intensity and type of prehabilitation are not yet standardised [7].

Evidence shows that prehabilitation improves patients’ quality of life [15] and reduces complication rates [16–19], mortality [17, 20], and hospital length of stay [21, 22]. Prehabilitation might be particularly cost-effective for high-risk patients [23]. However, evidence of its effectiveness for older people with frailty syndrome from randomised clinical trials is scarce, with only a few published trials [24–29]. In addition to prehabilitation’s (cost-)effectiveness, it is essential to know the factors that facilitate and hinder participation to adequately adapt prehabilitation programmes and successfully implement them into the healthcare system. Only a few studies have focused on facilitators and barriers to prehabilitation from the patients’ perspective [30]. In a recent mixed-methods systematic review including 23 studies [30], no study explicitly included frail patients.

This qualitative study aimed to use a theoretical model to capture the spectrum of physical, psychological, social, motivational, and other personal factors that facilitate and hinder participation in a prehabilitation programme prior to elective surgery from the perspective of older people with frailty syndrome and thereby inform future implementation.

## Methods

### Study design

This qualitative study was nested within the PRAEP-GO trial (NCT04418271), a multicentre randomised controlled trial currently investigating the (cost-)effectiveness of multimodal prehabilitation prior to elective surgery for elderly (pre-)frail patients in Germany [31, 32]. Following frailty screening, patients allocated to the intervention group participated in a shared decision-making conference followed by three weeks of individualised multimodal prehabilitation. Possible modalities included physiotherapy (exercise therapy, mobility or balance training), occupational therapy, speech and language therapy, nutritional counselling, psychosocial counselling and medical assessments as needed. Patients allocated to the control group received standard preoperative care. The primary endpoint is the change in the level of care dependency one year after the elective surgery [31]. Trial participants could additionally enrol in the accompanying research project, ANA-PRAEP-GO [33], which, amongst others, included this qualitative study.

Semi-structured, guided individual interviews were conducted to record and analyse the individual perspectives of the patients [34], and socio-demographic data were collected to describe the sample. The qualitative study was prospectively registered in the Open Science Framework (OSF) Registries (osf.io/xnbqc) on 18 February 2023 [35]. The study was reported using the Consolidated Criteria for Reporting Qualitative Research (COREQ) [36] (Appendix A).

### Ethics and data protection

The ethics committee of Charité – Universitätsmedizin Berlin granted ethical approval for both the PRAEP-GO trial (EA1/225/19) and the ANA-PRAEP-GO accompanying research project (EA1/266/20). For the present qualitative study, additional ethical approval was obtained from the ethics committee of Charité – Universitätsmedizin Berlin (EA1/272/22) on 1 February 2023. All participants were transparently informed in advance about the qualitative study and data protection regulations. Written informed consent was obtained before the interviews were conducted.

### Participants

We included participants of the PRAEP-GO trial who had been enrolled at Charité – Universitätsmedizin Berlin, a large university hospital in Berlin, between 30 June 2020 and 1 February 2023 and had been assigned to the intervention group. Consent to participate in the accompanying scientific programme (ANA-PRAEP-GO project) was a prerequisite. Table 1 provides an overview of all applied inclusion and exclusion criteria. More detailed information about the PRAEP-GO and ANA-PRAEP-GO trials is available from the published trial protocol [31] and registration records [32, 33].

**Table 1:** Inclusion and exclusion criteria

	Inclusion criteria	Exclusion criteria
PRAEP-GO trial *(31)*	- Age ≥ 70 years - Ability to give consent or having a legal guardian - Elective surgery planned - Expected duration of anaesthesia ≥ 60 min - Enrolled in statutory health insurance - Prefrail or frail on the basis of Fried et al. 2001 frailty phenotype (8)	- Severe heart disease (New York Heart Association grade IV) - Severe lung diseases (Global Initiative for Chronic Obstructive Lung Disease Grade IV) - Intracranial surgery - Palliative situation - Language barriers - Participation in another intervention study not authorised by the project management - Lack of consent to participate in the study
Present qualitative study	- Participants in the PRAEP-GO trial who were assigned to the intervention group - Participants in the accompanying ANA-PRAEP-GO programme with consent to be contacted regarding follow-up studies - Place of residence in Berlin or within a radius of about 35 km - Consent to be contacted by the interviewer	- None

### Recruitment

The interviewer (CSc) consecutively contacted eligible patients between 21 January 2023 and 6 March 2023 via telephone and informed them about the qualitative study. Prior to this contact, there was no relationship between CSc and the interviewees. Patients interested in participating in an interview were sent the study information and the informed consent in advance by email or post. Recruitment was based on convenience sampling [37] and was stopped after the eighth interview had been arranged. The maximum number of interviews that could be conducted had been determined in advance based on limited resources and a pre-defined time frame.

### Research team

The core research team consisted of CSc and TIF. CSc, a female postgraduate public health student with experience in qualitative research, conducted all interviews. CSc is a qualified occupational therapist with expertise working with older (pre-)frail people. Additionally, CSc has been working on various research projects at the Federal Institute for Occupational Safety and Health [38] since October 2021. TIF acted as the second coder of the interviews. TIF is a female postgraduate student of Public Health with a Bachelor’s degree in Medical Management and expertise in qualitative research, who at the time worked as a student assistant at the Institute of General Medicine at Charité – Universitätsmedizin Berlin. Coding was supervised by TR, a female post doctorate researcher with experience in qualitative research.

### Data collection

CSc conducted all interviews in the participants’ homes, with the option of having assistance from a familiar person. Before the interview began, verbal information about the study was provided with the opportunity to ask questions. Interviews were semi-structured and followed a pre-established interview guide (Appendix B). The interview guide was created following the methods by Helfferich 2014 [34], based on the literature on facilitators and barriers to prehabilitation [39–46]. It was piloted and tested for comprehensibility with three people over 65 who had previously participated in rehabilitation [47]. In addition to the closed and open-ended questions that constituted the interview guide, specific follow-up questions were asked that were adapted to the course of the interview. At the beginning of each interview, the socio-demographic data of the study participants were obtained in written format. After that, the interviews were recorded using a tape recorder without internet access. There were no repeat interviews.

### Data analysis

After each interview, the audio recordings were transcribed verbatim and analysed using the qualitative data analysis software MAXQDA Plus (version 22.5.0) [48]. Transcripts were not returned to participants. The data were analysed according to Kuckartz and Rädiker 2022 [49] using a content-structuring qualitative content analysis with a deductive-inductive approach, i.e., the data was structured in terms of content, analysed based on the structure, and categories were formed both deductively and inductively with a multi-stage process of categorisation and coding. In this study, a system of categories was developed and documented in a coding guide (available from the OSF project: osf.io/c2jx6/) to delimit the individual categories and to aid in clear coding [49]. This was reviewed for appropriateness during the entire process and revised several times.

After the complete coding of the data material by CSc, an independent coding was carried out by TIF using the coding guide. This served to check the quality of the coding [49]. The coded texts were then compared using MAXQDA (consensual coding) [49]. According to Kuckartz and Rädiker 2022 [49], disagreements were resolved based on the coding guide. Participants were not invited to provide feedback on the findings.

Subsequently, MAXQDA was used to create a topic matrix for each subcategory, in which all text passages of a category were summarised. A category-based analysis was carried out along this matrix. Socio-demographic data were analysed descriptively.

### Data synthesis

**Fig. 1:**
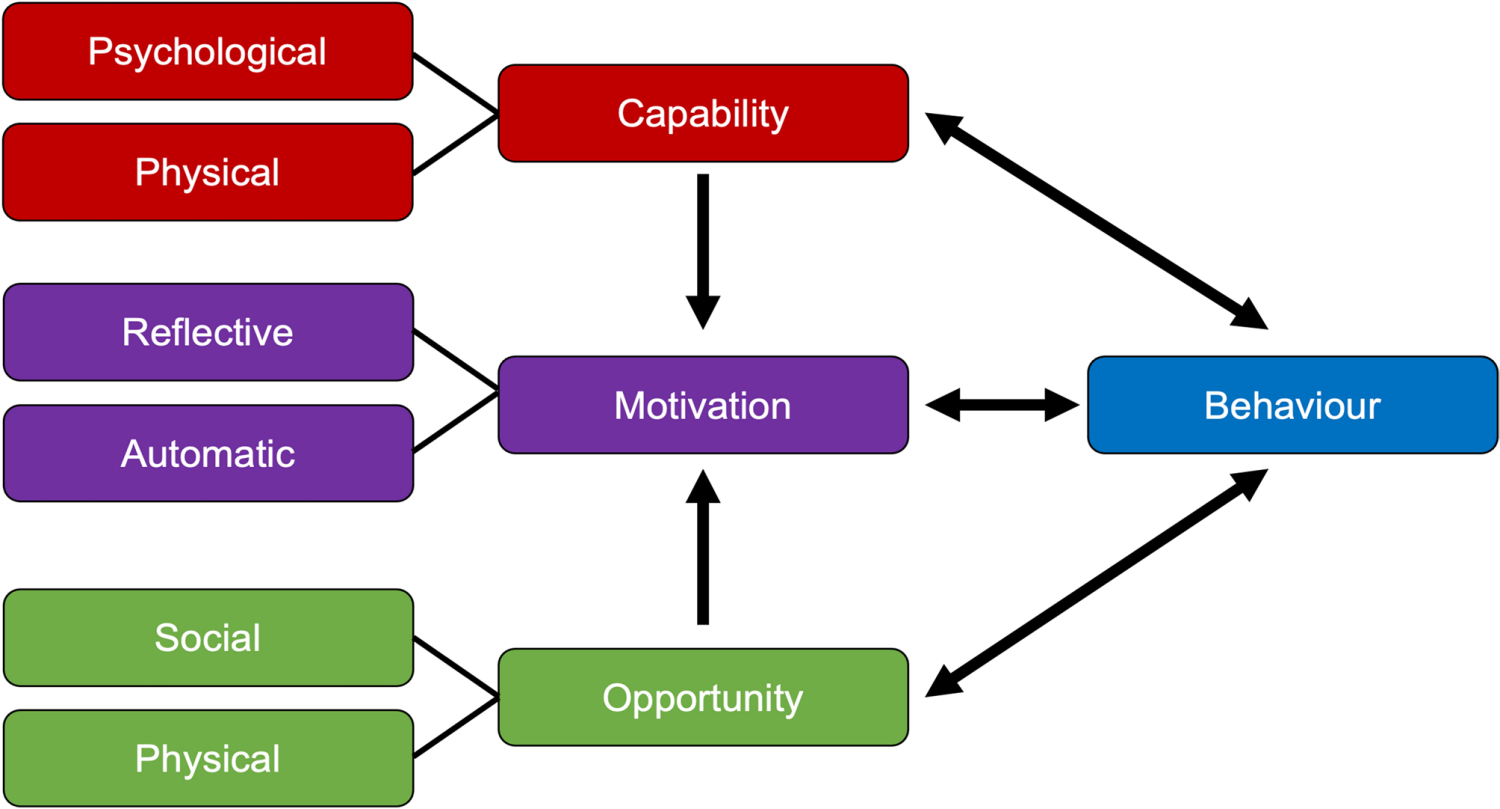
COM-B Model, own figure based on Michie et al. 2011 [52]

The COM-B model was used as a theoretical framework for categorising the facilitating and hindering factors mentioned by the patients. The COM-B model consists of three components: Capability, Opportunity, and Motivation [50], each of which is subdivided into two characteristics and interacts with Behaviour (Fig. 1). The model is used to analyse and understand behaviour - in this case, participation in prehabilitation - and to identify all the external and internal mechanisms that may be involved in behaviour change. Understanding the factors influencing behaviour change is vital for developing effective interventions [51]. All three components are necessary for a specific behaviour to occur [52]. The advantages of using this model are that it is an evidence-based, widely used and accepted behavioural science framework. It is simple and clear, helping to quickly identify key barriers and facilitators. Furthermore, it is comprehensive and holistic because it considers internal and external influences, as well as conscious decisions and habitual behaviours. While other theoretical frameworks, such as the Theoretical Domains Framework, were considered, the COM-B model was ultimately selected due to its conceptual clarity, practical applicability, and prior use by the research team in related contexts. This supported a consistent and structured approach to data analysis.

## Results

### Study sample

**Fig. 2:**
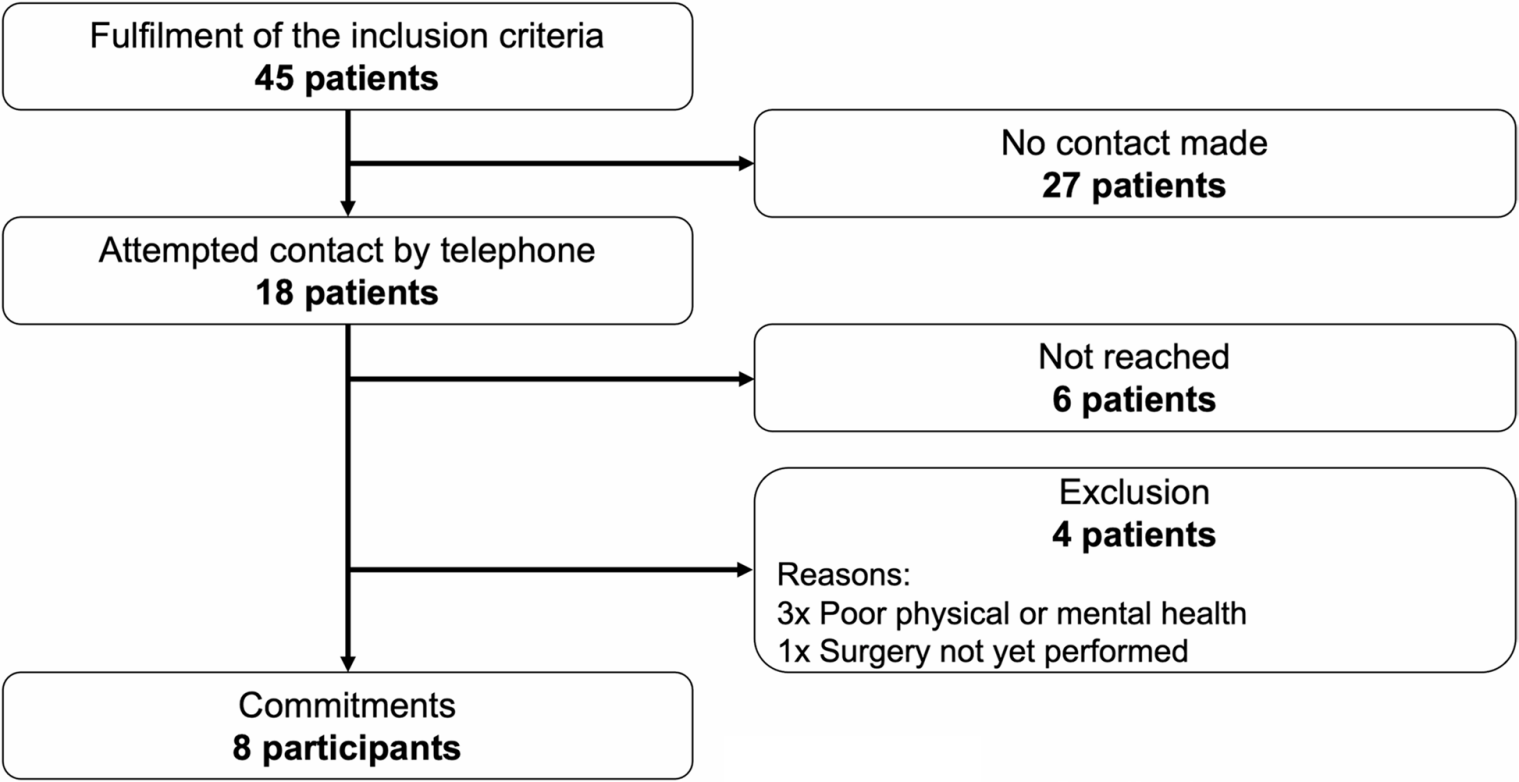
Flowchart for the recruitment of participants

A total of 45 patients fulfilled the inclusion criteria within the recruitment period. After consecutively contacting 18 patients, eight agreed to participate in this qualitative study, and enrolment was stopped (Fig. 2). Between 26 February and 13 April 2023, eight interviews were conducted in the participants’ homes, and the mean duration of the interviews was 20 min (standard deviation: 3 min). In seven interviews, only the interviewer and the study participant were present. In one interview, a person close to the patient was also present in the room but did not take part in the interview.

The participants’ characteristics are shown in Table 2. The mean age of the interviewees was 78.5 years (standard deviation: 3.0 years). Of note, all interviewees happened to have an indication for an orthopaedic surgery, although other types of surgeries were included in the PRAEP-GO trial as well.

**Table 2:** Participant characteristics (*N* = 8)

Participant characteristics	*N*	%
*Gender*		
Female	5	62.5
Male	3	37.5
Diverse	0	0.0
*Type of surgery*		
Hip surgery	4	50.0
Knee surgery	1	12.5
Spinal surgery	3	37.5
*Dependent on long-term care before surgery*		
Yes	2	25.0
No	6	75.0
*Participated in prehabilitation*		
Yes	8	100.0
No	0	0.0
*Setting of prehabilitation*		
Outpatient	7	87.5
Inpatient	1	12.5
*Previous knowledge about prehabilitation*		
Yes	0	0.0
No	8	100.0
*Duration of prehabilitation*		
3 weeks (as planned in PRAEP-GO)	5	62.5
< 3 weeks	1	12.5
unclear	2	25.0

### Course of prehabilitation

All participants received prehabilitation daily from Monday to Friday. According to the participants, the therapeutic sessions varied from 30 minutes to three hours per day. All participants underwent exercise therapy, with six participants using exercise machines. Both individual and group therapy formats were used. Seven participants were offered a nutritional counselling programme, three of whom reported taking part in it and four declined. All participants attending outpatient prehabilitation (*N* = 7) used taxi vouchers offered to travel to and from the prehabilitation facility. Surgery and prehabilitation had taken place on average just under eight months (range: 2–12 months) before the interview.

### Participants’ previous experience

All participants had experience with physical activity prior to prehabilitation. The extent of this varied: five participants had previously been active in sport, while three were familiar with everyday movements, such as walking or cycling. The majority (*N* = 6) had experience with one or more of the modalities (e.g., physiotherapy), although in some cases, this was many years ago (*N* = 3). The term “prehabilitation” (in German: “Prähabilitation”) was problematic for all participants to pronounce and, in some cases, unknown. The concept of the frailty syndrome was also unclear to all participants. The majority (*N* = 7) recommended prehabilitation or would participate in it again, while one person did not make a statement on this.

### Facilitating factors and barriers to participation in prehabilitation

A total of 32 facilitating factors and 25 barriers were identified (Table 3). All facilitators and barriers are listed, regardless of the number of times each was mentioned. The number of participants whose responses were mapped to each theme are also reported.

#### Psychological capability

In the context of psychological capability, two topics were identified as facilitating factors: First, knowledge of the benefits of prehabilitation after the concept was explained, which contributed to the decision to participate. Second, the availability of sufficient general information about prehabilitation. This included explaining the content and objectives of prehabilitation in a detailed and understandable way so that the participant was aware of what to expect and that the information provided was perceived as sufficient.

*“[…] I knew beforehand what to expect.” (Participant 2)*.

In contrast, the fact that the concept of prehabilitation was unknown until it was explained to patients and prior to inclusion in the PRAEP-GO trial was identified as a barrier. In addition, the concept of frailty syndrome was unknown to the eight participants, despite it being covered in the PRAEP-GO trial’s informed consent process, which prevented the potential benefit of information about it. Limitations in cognitive functions, e.g. memory loss, which resulted in not being able to remember information, were identified as another barrier.

*“I’ve read it now, but to be honest, I’m not quite sure what you mean by frailty syndrome." (Participant 2).*

#### Physical capability

The participants’ physical condition affected the decision to start or continue with prehabilitation. Facilitating factors were sufficient physical resources to carry out the physical modalities of prehabilitation and the prospect of overcoming existing physical deficits through participation.

*“Participant 5: […] not at all for six months. Almost didn’t get out of the apartment at all. Couldn’t walk up the stairs, nothing. Interviewer: So, did your physical condition also make you agree to this? Participant 5: Yes.” (Participant 5).*

Barriers relating to physical capability included physical limitations that prevented participants from participating in individual exercises during prehabilitation or endurance limitations. A key factor was pain, which prompted participants to cancel individual exercises or demand to be operated on as soon as possible and not to wait any longer than necessary for the surgery. In addition, acute and chronic illnesses were mentioned to be a barrier to participation.

*“I just couldn’t do some of the exercises because of my disability […].” (Participant 4).*

#### Social opportunity

Six facilitating factors were identified in this area. Healthcare professionals played a crucial role in the following facilitating factors: Recommendation of prehabilitation by the medical staff, a general attitude of trust towards the healthcare staff, and individualised care, which made participants feel heard and taken care of. Furthermore, the consistency of staff, their friendliness and competence positively affected continuing prehabilitation. Support from family members, dialogue with other patients and personal contact with the coordinators were also beneficial.

*“[…] the coordinator there was very helpful, and she also gave a lot of information and responded to my concerns […]. I really felt very well looked after there […].” (Participant 1).*

Barriers regarding social opportunities included when healthcare professionals discouraged participants from performing specific exercises, resulting in some modalities of prehabilitation not being carried out. The lack of individual support, e.g., when participants’ wishes and capabilities were not considered or they felt not taken seriously, resulted in participants cancelling appointments or lack of understanding. Changing healthcare professionals, lacking trust and lacking social support from the participants’ environment were also identified as barriers to participating in prehabilitation. In addition, it was a barrier when patients expected disadvantages for their relatives due to their absence as a result of participating in prehabilitation.

*“[…] But partly it [the programme] wasn’t catered to the patient*,* i.e. to me*,* so that I was not supposed to rotate my spine*,* but I was told that I should participate*,* so I had problems because of that and had to stop that [the session] and things like that […].” (Participant 4)*.

#### Physical opportunity

Several facilitating factors were identified relating to physical opportunity. These included organising prehabilitation and transportation in a way that participants did not have to worry about anything and the individual modalities of prehabilitation being appropriate to the participant’s capabilities. Furthermore, patients valued the existence of precise guidelines within the prehabilitation programme and when there were different choices for certain aspects of prehabilitation. The voluntary nature of the programme and the possibility of deciding on the intensity of the exercises oneself were also considered important, as well as the handout of materials with comprehensive information on the prehabilitation process. Additionally, providing transport by taxi vouchers to the prehabilitation facility and back was identified as a facilitator. Both covering the costs and organising travel to and from the prehabilitation centre were crucial, as well as saving time compared to public transport. The time aspect was also mentioned concerning the participants’ available time. Participants’ willingness to participate increased if they felt they had no other commitments or tasks. The direct transition from prehabilitation to surgery and the availability of prehabilitation places were also facilitating factors. The shared decision-making conference, home visits, a longer duration of prehabilitation or a greater variety of individual modalities of prehabilitation were also identified to have a facilitating effect.

*“[…] [I] was picked up at the door there, picked up again at the door and brought home, so it all worked out wonderfully. [...]" (Participant 6).*

Barriers relating to external opportunities included when individual modalities (e.g. nutritional counselling, exercise machines …) of prehabilitation were unavailable at one of the prehabilitation centres, a lack of choice of setting or facility, difficulty in the local accessibility of prehabilitation centres, and poor facilities. Another barrier related to staffing problems. This included prehabilitation staff lacking German language skills and a shortage of specialised staff. Participants’ private obligations and the resulting lack of time made them reject specific modalities of prehabilitation. Having a fixed surgery date or if the costs of prehabilitation would not be fully covered was also cited as a barrier to participation in the (entire) prehabilitation programme. Furthermore, one participant commented on the fact that they prefer other forms of contact (personal contact, telephone contact) over digital contact, in which case it would be a barrier if the shared decision-making conference and prehabilitation programme were purely based on digital contacts. Finally, restrictions due to the COVID-19 pandemic, such as nationwide lockdowns, resulted in fewer offers and delays.

*“I couldn’t afford it if I had to pay myself.” (Participant 8)*.

#### Reflective motivation

Positive expectations of prehabilitation were reported as a facilitating factor. These included the idea that participation in prehabilitation would make the surgery easier to tolerate and improve postoperative mobility and the attitude that the programme could be helpful in general. Positive experiences within the prehabilitation programme were also beneficial, e.g., the belief that the programme was valuable and essential for the participants’ own treatment and served as preparation for rehabilitation. Previous positive experiences with exercise and specific goals regarding the programme motivated participants to participate in prehabilitation.

*“[…] it can actually only help you so that the muscles stay a little stronger and are not completely gone." (Participant 3).*

Lacking clarity about the meaning of prehabilitation and participants’ lack of self-confidence presented barriers to reflective motivation.

*“[…] Climbing stairs*,* I never have to climb stairs here at home because I chose to live in this apartment. However*,* I was supposed to climb stairs there*,* so I just said: ‘No*,* I can’t*,* and I won’t do that […].’” (Participant 4)*.

#### Automatic motivation

An open-minded attitude, a high level of intrinsic motivation and the belief that participation would be advantageous were identified as facilitation factors for participation in prehabilitation.

*“So*,* I also wanted this intervention on my initiative […].” (Participant 4)*.

However, participants’ attitudes were also identified as a barrier to participation in prehabilitation, as well as fear of not understanding technical terms or explanations.

*“[…] if I don’t want to do it*,* then I won’t do it.” (Participant 1)*.

**Table 3:** Facilitators and barriers to participation in prehabilitation by behaviour change component

COM-B Component	Facilitators (*N* of participants who mentioned them)	Barriers (*N* of participants who mentioned them)
Psychological capability	(*N* = 6) • Knowing the benefits of prehabilitation (*N* = 2) • Receiving sufficient information about prehabilitation (*N* = 4)	(*N* = 8) • Lacking knowledge about the concept of prehabilitation (*N* = 8) • Lacking knowledge about the frailty syndrome (*N* = 5) • Impairment of cognitive function (*N* = 1)
Physical capability	(*N* = 6) • Own physical condition (*N* = 5) • Absence of pain (*N* = 3) • Age (*N* = 1)	(*N* = 5) • Limitation of physical abilities (*N* = 4) • Presence of pain (*N* = 2) • (Co-)morbidities (*N* = 3)
Social opportunity	(*N* = 8) • Recommendation from healthcare professionals (*N* = 5) • Personalised advice from healthcare professionals (*N* = 7) • Healthcare professionals remaining the same (*N* = 1) • Dedicated healthcare professionals (*N* = 6) • Support from family members (*N* = 4) • Interaction with other participants (*N* = 1) • Personal contact (*N* = 1)	(*N* = 4) • Healthcare professionals advising against participating (*N* = 1) • Lack of personalised care by healthcare professionals (*N* = 2) • Changing healthcare professionals (*N* = 1) • Lack of social support (*N* = 1) • Family members expecting disadvantages (*N* = 1)
Physical opportunity	(*N* = 8) • Good organisation (*N* = 4) • Adequate offers (*N* = 6) • Variety of offers (*N* = 4) • Specific guidelines (*N* = 4) • Possibility of choice (*N* = 7) • Availability of information materials (*N* = 3) • Transportation service (*N* = 5) • Patient has time to participate (*N* = 2) • Direct transition from prehabilitation to surgery (*N* = 2) • Capacity at prehabilitation centres (*N* = 1) • Shared decision-making conference (*N* = 1) • Home visits (*N* = 1) • Longer prehabilitation period (*N* = 1)	(*N* = 8) • Lack of offers (*N* = 2) • Lack of choice (*N* = 2) • Poor local accessibility of the prehabilitation facility (*N* = 1) • Poor facilities, e.g., state of equipment (*N* = 1) • Personnel problems (*N* = 2) • Participants having private obligations (*N* = 1) • Fixed date for surgery (*N* = 1) • Financial burden (*N* = 1) • Digital formats (*N* = 1) • Restrictions due to the COVID-19 pandemic (*N* = 2)
Reflective motivation	(*N* = 8) • Positive expectations regarding prehabilitation (*N* = 5) • Positive experiences with prehabilitation (*N* = 5) • Positive experience with exercises (*N* = 1) • Precise objectives (*N* = 2)	(*N* = 2) • Uncertain about the need for individual elements of prehabilitation (*N* = 2) • Lack of self-confidence (*N* = 1)
Automatic motivation	(*N* = 8) • Open-minded attitude (*N* = 5) • Intrinsic motivation (*N* = 3) • Not worried about disadvantages (*N* = 7)	(*N* = 3) • Inflexible attitude (*N* = 3) • Fear of not understanding certain aspects (*N* = 1)

## Discussion

The present study identified a total of 32 facilitators and 25 barriers to participation in prehabilitation prior to orthopaedic elective surgery through individual interviews with eight participants with frailty syndrome from the PRAEP-GO trial. The most frequently mentioned facilitators were the physical condition of participants, competent, accessible and familiar healthcare professionals, a well-organised prehabilitation programme with adequate offers, the possibility of choice and no concerns that prehabilitation could be disadvantageous or harmful. Frequently mentioned barriers comprised a lack of knowledge about prehabilitation and the frailty syndrome, limitations in physical functioning, the presence of (co-)morbidities and an inflexible attitude.

Compared to existing literature, an Australian study of 103 adult patients awaiting major gastrointestinal and urological cancer surgeries found that the majority were unaware of the concept of prehabilitation prior to planned surgery [53]. This was the case for all participants in the present study. This is probably because prehabilitation is not included in the standard care covered by statutory health insurance in Germany. However, the term is already included in some guidelines [54] and the websites of selected hospitals [55, 56]. Therefore, establishing this term in routine care via sufficient patient information is essential. The implementation into standard care could be an important step in this manner.

In the present study, the recommendation of health professionals for prehabilitation was critical in facilitating the decision to participate in the intervention. This finding is in line with that of Waterland et al. (2021), that patients were likely to participate in a prehabilitation program if it was recommended by their healthcare professional [53]. A medical consultation provides an excellent opportunity to discuss preoperative interventions and explain their potential benefits. Detailed, individualised advice is crucial for older people, who may have special requirements due to changing needs [57]. Subsequently, a recommendation by healthcare professionals and comprehensive information about the opportunities and benefits of prehabilitation may be essential to motivate patients to participate.

A cross-sectional study published in 2022 identified transportation problems, lack of time and parking costs as the main barriers to participation in prehabilitation [58]. Due to the organisation of transportation and coverage of treatment and travel costs in the PRAEP-GO trial, these factors were not perceived as barriers in the present study. Still, they were highlighted as facilitating factors, so they should be continued in future programmes.

As there is evidence that prehabilitation can benefit older (pre-)frail patients [3, 7, 13, 59–61], it seems reasonable to allow sufficient time for it. van der Zanden et al. 2021 [45] found that both patients and professionals considered it possible to postpone surgery if it would not affect the prognosis [45]. This largely depends on the underlying disease and its natural progress, e.g., slowly progressing knee osteoarthritis versus a more rapidly processing type of cancer. Nevertheless, a systematic review of waiting times for surgery for colorectal cancer came to the conclusion that there is no association between treatment delay and reduced overall survival in colon cancer patients [62], which underlines the feasibility of postponing elective surgery. In our interviews, the pain was explicitly mentioned as a reason to abort the prehabilitation programme to get the surgery sooner. Systematic knowledge about pain as a potential barrier in prehabilitation or rehabilitation is scarce. Paech et al. 2012 demonstrated that pain is a motivational factor for starting rehabilitation, while it was observed as a barrier to maintaining higher levels of physical activity six months after rehabilitation for those still experiencing pain [63]. Additionally, higher pain levels during exercises were identified as a barrier in a systematic review by Jack et al. 2010 [64]. Therefore, adequate pain management to assist participation in exercise therapy seems to be an essential factor for prehabilitation, acknowledging that some kind of exercise may be restricted due to the underlying disease for which surgery is planned. Furthermore, it may also be beneficial to discuss with patients what constitutes acceptable pain and their expectations of treatment.

Support from healthcare professionals and social support were found to be important facilitators. This finding is supported by a qualitative study by Agasi-Idenburg et al. 2020 [65]. The patients, the informal carers, and the healthcare providers interviewed in their study stated that practical and emotional support about the training was beneficial [65]. This shows that it is not only the patient’s perspective that may be of interest in implementing a prehabilitation programme but also that of family members and other stakeholders. A qualitative study by Fuchs et al. 2024 identified facilitators and barriers from healthcare professionals to implementing prehabilitation. They demonstrated that sufficient organisational infrastructure, human resources, access to knowledge, and an adaptable and individualised programme design could be beneficial for successfully implementing prehabilitation [66]. As the views of patients and healthcare professionals can differ [65], this opens up further areas for research.

Patient motivation was a component where facilitators and barriers have been identified and may largely determine patients’ adherence to prehabilitation. Agasi-Idenburg et al. 2020 report that compliance with prehabilitation varies, with values ranging from 16% to 97% [65]. However, it was particularly low in older people with (pre-)frailty [24, 29]. To increase adherence, prehabilitation programmes should be tailored to patient’s individual needs [45, 67, 68]. This also became evident in the present study, where participants mentioned adequate offers, a variety of offers and the possibility of choice as facilitating factors, thereby underlining the need for personalised prehabilitation programmes. This fact was supported by a realist review that considered facilitators and barriers to implementing prehabilitation for frail patients into routine healthcare [69].

Prehabilitation programmes’ individual modalities and settings may also play an essential role in participation. In the present study, participants had a positive attitude towards physical exercise and expressed that they found it helpful. The participants had more reservations about nutritional counselling, the shared decision-making conference and the psychosocial counselling. Future research should evaluate potential reasons for that and optimal combinations of modalities. In addition, future studies should compare home-based prehabilitation programmes with face-to-face prehabilitation programmes or a mixture of both. Barnes et al. 2023 [68] examined the facilitators and barriers of a home-based prehabilitation programme in their qualitative study. It concluded that it was feasible, manageable and easy to follow for older people with (pre-)frailty. Wang et al. 2021 [70] developed a mobile app as a tool to support a multidisciplinary prehabilitation protocol. However, the capabilities and limitations of older patients regarding digital technologies must be considered.

In light of the key findings from this qualitative study, the following aspects should be considered when designing future prehabilitation programmes for elderly (pre-)frail patients undergoing orthopaedic surgery: First, adequate patient selection is crucial, since participation in prehabilitation requires a certain level of physical capability in order to benefit from it. For patients with severe osteoarthritis, for example, this means that their symptoms, such as pain and stiffness, must be adequately managed to enable them to participate. At the same time, patients who consider themselves to be physically fit may not see the need to participate in a time-consuming programme. Secondly, our results emphasise the importance of offering patients the opportunity to choose which prehabilitation modalities they wish to engage with, and how. This tailored approach was valued by patients and may improve their motivation, engagement and programme retention. However, as there are currently no defined standards for the duration, frequency and modalities of prehabilitation programmes for (pre-)frail surgical patients, incorporating this flexibility may further complicate trial design. Thirdly, our participants had limited knowledge about prehabilitation and frailty syndrome, as both concepts are not yet widely embedded in routine clinical care. Instead, elderly patients tend to attribute symptoms of frailty, i.e. unintentional weight loss, exhaustion, muscle weakness, slow walking speed, and low physical activity, to their age. Consequently, many patients believe that this is the new status quo and that it cannot be changed. However, targeted interventions, such as prehabilitation, can improve these symptoms. This emphasises the need for comprehensive, tailored information strategies to be an integral part of future prehabilitation programmes for (pre-)frail patients. Lastly, (pre-)frail patients require additional support to participate in prehabilitation (e.g. transport to and from the facilities) and their relatives or carers should be closely involved. It may even be necessary to organise care for a spouse while the patient attends prehabilitation, particularly if they are also elderly and frail.

### Strengths and limitations of the study

This is the first qualitative study conducted in Germany of older (pre-)frail patients who have received prehabilitation before a planned surgery, intending to strengthen patients’ role in the implementation of prehabilitation. The synthesis of the facilitators and barriers using the COM-B model, which represents an overarching behavioural model, allowed us to comprehensively cover many different issues, which is the basis for developing evidence-based interventions [50]. In addition, the coding and categorisation in the COM-B model were carried out by two researchers independently to achieve a high level of internal validity.

However, some limitations should be considered when interpreting the results. First, the study’s sample size was very small, so although theoretical saturation was aimed for [47], it was not achieved because new aspects had been raised in each interview. Furthermore, because of the study-specific inclusion criteria (e.g., participation in the ANA-PRAEP-GO accompanying research project), the results are not representative of all patients allocated to the prehabilitation group. Moreover, all participants interviewed agreed to participate in prehabilitation, indicating that they were highly motivated and that the views of patients who had refused or cancelled prehabilitation could not be included. Due to the method of convenience sampling without stratification, only participants with orthopaedic conditions happened to be interviewed in this study. It is, therefore, possible that the facilitators and barriers identified may not be readily transferable to patients with other underlying diseases. In addition, although frailty was assessed as part of the broader PRAEP-GO trial, it was not possible to link the results from the frailty screening to the interviewees due to the data protection and confidentiality regulations within the trial. Other limitations include the timing of the interviews. In some cases, the prehabilitation occurred twelve months ago, which may have biased their answers due to missing or altered memories. Finally, only patients in and around Berlin, a major city with over 4 million inhabitants, were surveyed, so it is possible that factors applying to patients from rural areas were not captured.

## Conclusion

This qualitative study provides an overview of the factors that facilitated and hindered participation in a prehabilitation programme for older patients with frailty syndrome prior to elective surgery in Germany. Many factors have already been considered in the prehabilitation programme of the PRAEP-GO study and were confirmed to facilitate participation. Nevertheless, barriers must be addressed when developing and implementing such a programme into routine care. For example, future prehabilitation programmes should be even more tailored to the frail patient’s needs while being cost-effective and feasible. This could be realised by incorporating digital solutions and expanding on home-based modalities.

## Supplementary Information

Supplementary Material 1.

## Data Availability

Due to the data protection regulations of the study, no raw data can be made available. However, a complete coding guide, including interview quotations, is available from the OSF project (osf.io/c2jx6/).

## Abbreviations

ANA-PRAEP-GO: Analysis of Frailty Syndrome Within the Framework of the Innovation Fund Project PRAEP-GO
COM-B: Capability, Opportunity, Motivation - Behaviour
COREQ: Consolidated criteria for reporting qualitative research
PRAEP-GO: Prehabilitation of Elderly Patients With Frailty Syndrome Before Elective Surgery
