# Supplementary material for: Facilitators and barriers to participation in prehabilitation prior to orthopaedic elective surgery – a qualitative study with elderly (pre-)frail patients
Source: BMC Geriatr. 2025 Nov 4;25:845. doi: 10.1186/s12877-025-06592-3 (PMC12584233; doi:10.1186/s12877-025-06592-3)
Supplement: Supplementary file 1 — Supplementary Material 1. [file 12877_2025_6592_MOESM1_ESM.pdf]

Appendix A: COREQ: 32-item checklist

No. Item	Guide questions/description	Reported on Page #
**Domain 1: Research team and reflexivity**		
Personal Characteristics		
1. Interviewer/facilitator	Which author/s conducted the interview or focus group?	8
2. Credentials	What were the researcher's credentials? E.g. PhD, MD	8
3. Occupation	What was their occupation at the time of the study?	8
4. Gender	Was the researcher male or female?	8
5. Experience and training	What experience or training did the researcher have?	8
Relationship with participants		
6. Relationship established	Was a relationship established prior to study commencement?	7
7. Participant knowledge of the interviewer	What did the participants know about the researcher? e.g. personal goals, reasons for doing the research	7
8. Interviewer characteristics	What characteristics were reported about the interviewer/facilitator? e.g. Bias, assumptions, reasons and interests in the research topic	7
**Domain 2: study design**		
Theoretical framework		
9. Methodological orientation and Theory	What methodological orientation was stated to underpin the study? e.g. grounded theory, discourse analysis, ethnography, phenomenology, content analysis	9
Participant selection		
10. Sampling	How were participants selected? e.g. purposive, convenience, consecutive, snowball	7
11. Method of approach	How were participants approached? e.g. face-to-face, telephone, mail, email	7-8
12. Sample size	How many participants were in the study?	11
13. Non-participation	How many people refused to participate or dropped out? Reasons?	11
Setting		

14. Setting of data collection	Where was the data collected? e.g. home, clinic, workplace	8
15. Presence of non-participants	Was anyone else present besides the participants and researchers?	8
16. Description of sample	What are the important characteristics of the sample? e.g. demographic data, date	12
Data collection		
17. Interview guide	Were questions, prompts, guides provided by the authors? Was it pilot tested?	8
18. Repeat interviews	Were repeat interviews carried out? If yes, how many?	9
19. Audio/visual recording	Did the research use audio or visual recording to collect the data?	9
20. Field notes	Were field notes made during and/or after the interview or focus group?	9
21. Duration	What was the duration of the interviews or focus group?	11
22. Data saturation	Was data saturation discussed?	25
23. Transcripts returned	Were transcripts returned to participants for comment and/or correction?	9
**Domain 3: analysis and findings**		
Data analysis		
24. Number of data coders	How many data coders coded the data?	9
25. Description of the coding tree	Did authors provide a description of the coding tree?	OSF materials (https://osf.io/c2jx6/)
26. Derivation of themes	Were themes identified in advance or derived from the data?	9
27. Software	What software, if applicable, was used to manage the data?	9
28. Participant checking	Did participants provide feedback on the findings?	9
Reporting		
29. Quotations presented	Were participant quotations presented to illustrate the themes/findings? Was each quotation identified? e.g. participant number	14-18
30. Data and findings consistent	Was there consistency between the data presented and the findings?	11-20
31. Clarity of major themes	Were major themes clearly presented in the findings?	13-20
32. Clarity of minor themes	Is there a description of diverse cases or discussion of minor themes?	13-20

N/A = not applicable

Appendix B: Interview guide

Greeting and introduction of the interviewer

Hello, my name is Carina Pfab. I am a student at the Berlin School of Public Health and will conduct the 20-to-40-minute interview with you.

Thank you, topic, aim of the interview

Thank you very much for agreeing to take part in the survey on the topic of "facilitating and hindering factors in participating in prehabilitation". I am writing my master's thesis on this topic. Your serve the goal of better designing prehabilitation programmes for elderly persons with frailty syndrome prior to surgery. In addition, the results may help to support the implementation of prehabilitation into the standard care of the statutory health insurance.

Voluntariness, confidentiality and data protection

Before starting, I would like to remind you that your participation is completely voluntary and all answers will be treated confidentially. As described in data protection declaration, paperbased data will be kept locked at the Technical University of Berlin and no one will be identified by name in the evaluation of the survey. In addition, all personal details (e. g. places and personal names) will be deleted in accordance with data protection regulations, so that it is virtually impossible to draw any conclusions about your person. With your consent, I record the interview and take notes. If you are uncomfortable with certain topics or individual questions, you do not have to answer them. Just let me know and we can skip them.

The procedure

At the beginning I ask you some personal questions. I write down the answers on paper. These will only be used in summarized form and will serve to describe all the people interviewed. Then I start the recording and begin the actual questioning about your experiences of prehabilitation or the factors why you were not able to do it (completely).

Do you have any questions for me before the interview starts?

Questions for the informal field notes

Then I start with the personal questions. I will start the audio recording afterwards.

1. How old are you?
2. What gender do you identify with?
3. Did you have a level of care before the operation? If so, do you know which one?
4. What kind of surgery did you receive?

Thank you very much for your answers. The main part of the interview follows. For this, I will now start the audio recording. Do you agree?

________________________*Recording starts*______________________________

The recording is now running. Thank you again for agreeing to do the interview in person/by phone (select relevant). Your pseudonym is "TN..." [insert appropriate pseudonym].

General information about PRAEP-GO

- Have you received prehabilitation?
- Were you aware of the concept of prehabilitation before you participated in the new form of care PRAEP-GO?
- What experience did you have with physical activity and therapeutic treatments (e. g. self-exercise, rehabilitation) before the surgery?

Specific to prehabilitation

- *Only prehabilitated persons:* in what form was prehabilitation carried out for you (outpatient, (partial-)inpatient)?
    - Were you able to carry it out to the full extent?
- What thoughts, expectations and concerns did you have about prehabilitation beforehand?
    - In your opinion, how well informed were you about prehabilitation in advance?
- What factors influenced your decision to [not] participate in prehabilitation?
    - What impact had:

    - your state of health before the operation
    - structural conditions (e. g. travel distances)
    - financial aspects (e. g. travel costs)
    - social obligations (e. g. caring for relatives)
    - social support (e. g. family, friends)
    - professional accompaniment (e. g. by health workers)
    - psychological conditions (complaints, lack of interest)
    - temporal factors
    - the diagnosis of frailty syndrome
- What problems or challenges could you identify [prevented you from participating]?
    - What made participation difficult [prevented you from participating]?
    - *Only prehabilitated persons*: What made it difficult to complete?
    - *Only prehabilitated persons*: What bothered you about prehabilitation?
    - Only non-prehabilitated persons: What was the disadvantage of participating?
- *Only prehabilitated persons*: What did you find helpful about prehabilitation?
    - What resources were you able to draw on?
- In what way do you think prehabilitation [would have been] important for your treatment?
- *Only prehabilitated persons*: What improvements would you like to see in the implementation of prehabilitation?
- Only non-prehabilitated persons: What would motivate you to participate in prehabilitation?
- Would you have preferred a different [used] another form of prehabilitation?
    - How would a programme have been ideal for you?

Conclusion

Thank you for your time and for answering the questions.
Is there anything else you would like to add that has not come up so far or that seems important to you?

______________________*Recording ends*____________________________
